# Nutritional knowledge, attitude and practices among pregnant females in 2020 Shenzhen China: A cross-sectional study

**DOI:** 10.1016/j.pmedr.2023.102155

**Published:** 2023-02-18

**Authors:** Wei-Chuan Wang, Si-Mei Zou, Zan Ding, Jia-Ying Fang

**Affiliations:** a Department of Clinical Nutrition, University of Chinese Academy of Sciences Shenzhen Hospital, Shenzhen, Guangdong 518106, China; b Department of Nutrition and Food Hygiene, School of Public Health, Southern Medical University, Guangzhou 510515, China; c Social Health Management Center, University of Chinese Academy of Sciences Shenzhen Hospital, Shenzhen, Guangdong 518106, China; d The Institute of Metabolic Diseases, Baoan Central Hospital of Shenzhen, Shenzhen, Guangdong 518102, China; e Medical Department, Huadu District People's Hospital, Southern Medical University, Guangzhou 510800, China

**Keywords:** Knowledge, Attitude, Practice, Pregnancy, Nutrition

## Abstract

The theory of knowledge, attitude and practice (KAP) can well explain the whole process of pregnant females' practice to obtain balanced nutrition. However, the process of KAP works very differently in populations with different sociodemography. This study aims to investigate the sociodemographic determinants associated with pregnant females' nutritional KAP and find a way to locate the vulnerable pregnant females who would benefit most from intervention. A cross-sectional survey was conducted on pregnant females' KAP regarding food nutrition at the University of Chinese Academy of Science Shenzhen Hospital from December 2020 to February 2021. A total of 310 pregnant females aged 18–40 years were interviewed. And we assessed the influence of sociodemographic factors on KAP and built a model for screening the vulnerable group who would benefit most from intervention. The results showed that on nutritional knowledge and practice, only 15.2% and 47.3% were above 0.6 respectively, while 91% were above 0.75 on attitude. Age, Husband's Education Degree, Monthly Income of Family, Nutritional Knowledge and Nutritional Attitude were statistically significant predictors of the vulnerable group. There was a gap between knowledge (3.8% were good or above) and attitude (91% were good or above), attitude and practice (16.8% were good or above). Age, household registry, education level, monthly income, and nutrition knowledge were associated with nutrition practices. This study highlights that nutritional education interventions targeting certain populations may improve the conversion rate of nutrition practices, and presents a predictive model to locate the vulnerable group.

## Highlights

• A gap existed between nutritional knowledge, attitude and practice among pregnant.
• Shenzhen’s pregnant females had high attitude, but low knowledge and practice.
• Nutritional knowledge and socio-demographics were associated with practices.
• A visualized model was built to locate the low-nutritional-practices group.

## 1 Introduction

Pregnancy is a critical time in a woman's life, as pregnant females will experience many changes including physical and psychological changes during pregnancy (Perlen et al., 2013). To provide enough nutrients for the growing fetus as well as ensuring the mother's well-being, the demand for energy, macronutrients, and micronutrients will be largely increased. Females in developing countries are at risk of malnutrition and nutritional deficiencies during pregnancy, which will lead to adverse pregnancy outcomes, such as delayed fetal growth, premature birth, low birth weight, and maternal anemia (Conde-Agudelo et al., 2012, Abou Haidar and Bou Yazbeck, 2018).

The theory of “Knowledge, Attitude and Practice” is the most commonly used model to explain how personal knowledge and beliefs affect health behavior change (Kwol et al., 2020). This model starts with instructing health educators to publicize health knowledge and strengthen health attitude, so that patients are willing to take active preventive measures to prevent and cure diseases (Klahr and Kotovsky, 2013, Chiesi et al., 1979). For its effectiveness in the field of behavior change, this theory is also widely used in the fields of management and public health (Miller and Cassady, 2015). Such as the management of chronic diseases in the community.

Nutritional information on food labels may be an effective way to communicate nutritional information to consumers, as it is displayed in most packaged foods (Graham and Roberto, 2016, UMEH, 2015). However, because of consumers' level of knowledge, shopping habits, and their own financial ability, nutritional information on food labels is not well utilized. Knowledge and attitudes about nutrition are important factors in dietary habits and are therefore potential interventions for developing appropriate nutritional health plans for pregnant females. Nutrition education improves nutrition knowledge, thereby influencing attitudes and practices towards good nutrition (Saeidlou et al., 2016). However, sociodemographic factors have also been reported to influence the adoption of appropriate nutritional practices (Masuku and Lan, 2014).

Shenzhen is a highly developed city, a city of immigrants, but also a city with a poor level of medical care. Shenzhen contains a relatively large migrant population of women who are generally less educated, not having Shenzhen health insurance and less likely to visit a doctor when they are sick (Mou et al., 2009). They still work in Shenzhen when they are pregnant, but will return home to give birth. This specificity veils the current status of nutrition practices for pregnant women in Shenzhen. High physical mobility rates and low levels of health literacy amongst those floating population in Shenzhen are major public health issues (Public health services in Shenzhen, 2022). In Shenzhen, the ratio of residents to migrants is approximately 1:3 (Zhou et al., 2021). In the past 10 years, the results of 492,184 births analysis in Shenzhen Baoan showed that migrant women accounted for 87% of the total population but had a higher stillbirth rate (4.8 per 1000 births) than the permanent population (2.8 per 1000 births) (Ma and Zou, 2022). Specifically, the stillbirth rate in Shenzhen between 2010 and 2019 was found to be inversely correlated with the GDP per capita of maternal birthplace (Spearman's coefficient is −0.875) (Ma and Zou, 2022). According to the National Bureau of Statistics and the census, the permanent resident population of Shenzhen reached 17.56 million in 2020 alone, an increase of more than 4 million in just one year compared with 13.43 million in 2019. The influx of a large number of migrants into the city of Shenzhen in a short time has led to dynamic changes of demographic features and birthplace sources. In recent years, there are few reports studying pregnant females' nutritional knowledge, attitudes, and practices (KAP) in Shenzhen, and it is unclear what sociodemographic factors will influence KAP. Therefore, this study aimed to investigate nutritional KAP and determine their association with sociodemographic factors of pregnant females in Shenzhen. In addition, multiple regression attempts to identify the key populations that would benefit most from intervention in time, and improve the level of pregnant females' nutrition in Shenzhen (Masuku and Lan, 2014). Therefore, research is needed to identify the level of knowledge, attitude and practice of pregnant females and to analyze their relationship with sociodemographic indicators.

## 2 Materials and methods

### 2.1 Subjects and study design

The study obtained ethical approval number LL-KT-2021158 from the University of Chinese Academy of Science Shenzhen Hospital Research and Ethics Review Committee. From December 2020 to February 2021, a cross-sectional survey was conducted on pregnant females' knowledge, attitudes, and behaviors regarding food nutrition at the University of Chinese Academy of Science Shenzhen Hospital. Criteria for inclusion in the study: Shenzhen's permanent population, aged 18–40, and no other underlying chronic diseases before pregnancy. Since respondents with other chronic diseases will have different knowledge requirements for nutrition, they were not considered in this study due to their specificity. Therefore interviewees, diagnosed with diabetes or hypertension, or other basic chronic diseases before pregnancy, or endocrine-related diseases such as hyperthyroidism before pregnancy were excluded. We randomly select 2 obstetricians from the hospital. At the time of those two obstetricians' outpatient visits, the investigator surveyed each pregnant female who came to the clinic by convenience sampling. The investigators are nurses and dietitians who have undergone uniform training. They distribute questionnaires to pregnant females who are undergoing obstetric check-ups in the hospital and supervise the completion of survey subjects. Informed consent preceded all interviews.

This questionnaire measures the KAP developed by the Institute of Nutrition and Food Safety of China Center for Disease Control and Prevention (Boheng Liang, 2016). This questionnaire was selected because it was suitable for the population of this study and had good reliability and validity. The Cronbach's α of the whole questionnaire was 0.90, and the retest reliability of knowledge, attitudes, and practices were all above 0.8. In addition, the content validity was also good, with a mean correlation coefficient of 0.65 between each item score and the total score. The content and main indicators of the questionnaire are as follows:

(1) Basic information: Age, local Household Registration, Height, Gestational weeks, Times of Pregnant, Times of Birth, Times of Abortion, Weight before pregnancy, Current weight, Education Degree, Career, and Monthly income of Family.
(2) Knowledge survey of nutrition labeling and basic nutrition: knowledge of basic nutrients (7 questions in total) and knowledge about nutrition labeling (4 questions in total). There are 11 questions in total.
(3) Survey of attitudes and practice on nutrition labels: pregnant females' attitudes towards food nutrition labels, dietary behaviors, and whether to use nutrition labels when shopping, etc. There are 9 questions in total.

Scoring strategy: Single item worth 1 score, multiple choices all right worth 2 scores, part is right worth 1 score, and multiple choices answered wrongly worth 0 points.

### 2.2 Statistical analyses

R (version 3.5.0) was adapted for data analysis. To describe the sociodemographic variables and nutritional KAP, continuous variables were presented as mean ± standard deviation (SD) while nominal variables were presented as the number with a percentage. To explore the association between the sociodemographic variables and nutritional practice, Pearson’s chi-squared test was used for nominal variables, nominal-ordinal Chi-square for nominal and ordinal variables, and independent *t*-test for continuous variables. To assess the correlation between nutritional KAP and selected continuous sociodemographic factors, Pearson's product-moment correlation coefficient was applied. Univariate linear regression was used to find the potential factors associated with nutritional KAP. A stepwise algorithm was used select appropriate features from the demographic variables and to build a multiple logistic regression model to locate the persons with a low level of nutritional practice. A Venn plot was applied to demonstrate the common factors associated with nutritional KAP and a nomogram plot was used to visualize the logistical regression. Statistical significance was set at *P* value less than 0.05. If there are missing key variables, these samples were omitted to perform a complete case analysis.

## 3 Result

### 3.1 Summary of responses to nutritional knowledge, attitude, and practice-related questions

A total of 317 pregnant females were surveyed during the data collection period, 7 refused to accept to be interviewed, and a total of 310 valid questionnaires were collected, with a response rate of 97.7%. As shown in Table 1, the vast majority of pregnant women have positive attitudes toward nutrition, but have less knowledge about nutrition and fewer nutrition-related practices. The percentage of pregnant women with good and above attitudes was 91%, but the percentage with good and above knowledge and practice was 3.8% and 16.8%, respectively.

**Table 1:** Summary of nutritional knowledge, attitude, and practice scores obtained by respondents (N = 310).

Questionnaire	Total score	Mean ± SD	Bad(<60%)	Normal(60%–75%)	Good(75%–90%)	Excellent(>90%)
Nutritional Knowledge	22	9.5 ± 3.9	263(84.8%)	35(11.3%)	11(3.5%)	1(0.3%)
Nutritional Attitude	9	7.6 ± 1	9(2.9%)	19(6.1%)	240(77.4%)	42(13.6%)
Nutritional Practices	11	5.9 ± 2.6	162(52.3%)	96(31.0%)	31(10.0%)	21(6.8%)

According to Appendix 1, the pregnant women interviewed generally had weak knowledge, and only knew a few common knowledge. 75.2% (233) answered which food provides high-quality protein correctly or partially, and 91.9% (282) correctly answered what nutrients cause cramps during pregnancy if lacking. But there were some nutritional misunderstandings. About half wrongly believed that carbohydrates and sugars are one thing (58.4%, 181) and milk is iron-rich (47.4%, 147). Furthermore, most generally lack knowledge of nutritional dietary guidelines and related food nutrition labels. More than half, 56.1% (174), had not heard of the nutritional dietary guidelines, 63.9% (198) don't know food packaging is subject to mandatory labeling of food nutrition labels; and the vast majority, 92.9% (288), didn’t know the meaning of NRV.

In terms of nutritional attitudes in Appendix 2, 99.7% (309) of the interviewees recognized that nutrition knowledge, to some extent, is important, and 99% (307) were willing to change unhealthy eating habits. For food nutrition labeling, 99.3% (308) believed that it is necessary to display nutrition labels on foods, but 21.0% (65) believe that the information on food nutrition labels is partially trustworthy.

In terms of nutrition practice in Appendix 3, only 26.8% (83) do not eat fried smoked or carbonated food, and only 45% (140) often or always look at the food nutrition label when selecting food. Among the key nutritional practices for pregnancy, only 64.5% (200) obeyed taking folic acid-rich foods or folic acid supplements daily, and 72.9% (226) had milk or calcium tablets daily. And only 57.4% (178) had experienced changing food choices because of food labels.

### 3.2 Sociodemographic characteristics of participants

In this study, scores less than 60% in nutrition practice can be considered as the key population for intervention (KPI). To find out the population with low practice, univariate analysis was used between sociodemographic variables and KPI. Referred to Table 2. Knowledge and attitude were significantly associated with the practice. The lower the knowledge and the lower the attitude, the easier to be classified as KPI, and the *P* values are 0.002 and 0.018, respectively. In addition, nutrition practice is also related to the education level of the interviewee and her husband. The lower the educational background of pregnant females (*P* = 0.003) and their husbands (*P* = 0.003), the more likely they are to become KAP. Similar to the educational factors, it is also related to the husband’s occupation. When the husband’s occupation is teacher/medical staff, company employee or other stable job, the practice of respondents tends to be higher. In addition to the factors above, practice is also related to age, whether it is a local household registration, and family monthly income. Pregnant females who are older, having a higher monthly household income, and local registration tends to have better practices, with *P* values of 0.004, 0.004 and 0.049, respectively. Other sociodemographic indicators such as Times of Pregnant, Times of Birth, Times of Abortion, Gestational weeks, Weight increment by week and BMI cannot be considered statistically different from practice (*P* > 0.05).

**Table 2:** Sociodemographic characteristics of pregnant in Shenzhen 2020(N = 310).

Features, n(%)/Mean(SD)	KPI group(Nutritional Practices lower than 60%)		*P*
	No	Yes	
Local Household Registration			0.049*
Yes	37(25)	25(15.4)	
No	111(75)	137(84.6)	
Career			0.107
Teachers/Medical staff	15(10.1)	10(6.2)	
Employees of public institutions	5(3.4)	3(1.9)	
Company employees	55(37.2)	41(25.3)	
Worker	9(6.1)	17(10.5)	
Farmer	3(2.0)	4(2.5)	
Freelancer	29(19.6)	37(22.8)	
Unemployed or others	32(21.6)	50(30.9)	
Career Of Husband			0.030*
Teachers/Medical staff	5(3.4)	2(1.2)	
Employees of public institutions	9(6.1)	12(7.4)	
Company employees	71(48.0)	65(40.1)	
Worker	18(12.2)	41(25.3)	
Farmer	2(1.4)	2(1.2)	
Freelancer	37(25.0)	39(24.1)	
Unemployed or others	6(4.1)	1(0.6)	
Times of Pregnant			0.844
1	41(27.7)	53(32.7)	
2	60(40.5)	52(32.1)	
3	24(16.2)	33(20.4)	
≥4	23(15.5)	24(14.8)	
Times of Birth			0.292
0	44(29.7)	63(38.9)	
1	89(60.1)	80(49.4)	
≥2	15(10.1)	19(11.7)	
Times of Abortion			0.627
0	88(59.5)	89(54.9)	
1	34(23.0)	45(27.8)	
≥2	26(17.6)	28(17.3)	
Education Degree			0.003*
High school or blow	49(33.1)	74(45.7)	
Secondary or tertiary	64(43.2)	69(42.6)	
Undergraduate or above	35(23.6)	19(11.7)	
Husband’s Education Degree			0.003*
High school or blow	36(24.3)	60(37.0)	
Secondary or tertiary	76(51.4)	80(49.4)	
Undergraduate or above	36(24.3)	22(13.6)	
Monthly income of Family			0.004*
≤5000	24(16.2)	43(26.5)	
5000–7000	29(19.6)	39(24.1)	
7000–10000	34(23.0)	40(24.7)	
10000–20000	45(30.4)	24(14.8)	
>20000	16(10.8)	16(9.9)	
Nutritional knowledge			0.002*
Bad	115(77.7)	148(91.4)	
Normal	25(16.9)	10(6.2)	
Good	7(4.7)	4(2.5)	
Excellent	1(0.7)	0(0.0)	
Nutritional attitude			0.018*
Bad	1(0.7)	8(4.9)	
Normal	8(5.4)	11(6.8)	
Good	115(77.7)	125(77.2)	
Excellent	24(16.2)	18(11.1)	
Age	29.4(4.5)	27.9(4.3)	0.004*
Gestational weeks	28.1(9.8)	26.9(10.9)	0.306
Weight increment by week	0.3(0.6)	0.3(1.1)	0.998
BMI	22.7(6.1)	22.9(6.4)	0.776

### 3.3 Correlation between sociodemographic and KAP variables

**Fig. 1:**
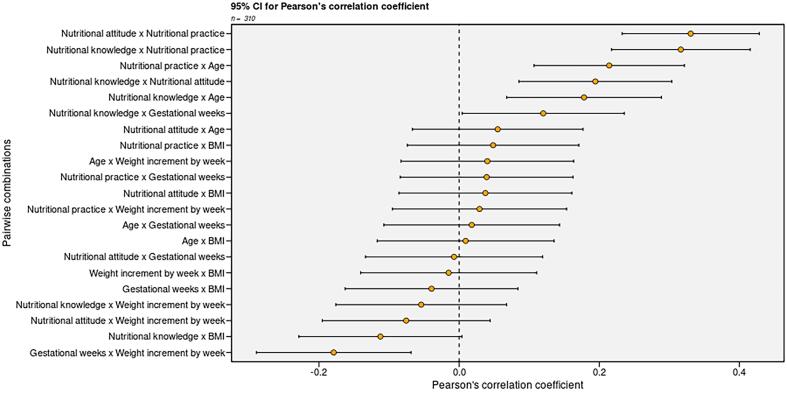
Results of Pearson’s correlation between nutritional KAP and sociodemographic variables.

Bivariate correlation analysis (Fig. 1) showed that gestational weeks (*r* = 0.12, *P* = 0.03), and age (*r* = 0.18, *P* = 0.002) were significantly positively correlated with knowledge. BMI (*r* = −0.11, *P* = 0.048) was significantly negatively correlated with knowledge; age (*r* = 0.21, *P* < 0.001) was significantly positively correlated with nutrition practice. Significant differences were observed between knowledge and attitude (*r* = 0.19, *P* < 0.001), knowledge and practice (*r* = 0.32, *P* < 0.001), and attitude and practice (*r* = 0.33, *P* < 0.001).

### 3.4 Univariate regression for predictors of nutritional knowledge, attitude, and practices

To find out the associated factors of knowledge, attitude and practice, univariate analysis was performed. According to Table 3, the factors affecting knowledge, attitude and practice were different. The sets of factors for knowledge and practice were quite large in number and mostly overlapped, but the set of factors for attitude was small. All the factors that were statistically significant from practice would also statistically significant from knowledge, namely Age, Local Household Registration, Times of Birth, Education Degree, Husband's Education degree, Monthly income of family and Career of Husband. There were 3 more factors （Gestational weeks, BMI, Career) that were statistically significant from knowledge but not from practice. Only 3 factors (Husband's education Degree, Monthly income of Family, Career) were statistically significant from the attitude dimension.

**Table 3:** Univariate analysis of factors associated with nutritional knowledge, attitude and practice.

Variable	Nutritional Knowledge		Nutritional Attitude		Nutritional Practice	
	*Coefficient (95 %CI)*	*P*-value	*Coefficient (95 %CI*	*P*-value	*Coefficient (95 %CI*	*P*-value
Age	0.154(0.059 to 0.250)	0.002	0.124(−0.012 to 0.037)	0.334	0.125(0.061 to 0.188)	<0.001
Local Household Registration						
Yes	ref		ref		ref	
No	−2.891(−3.925 to −1.856)	<0.001	0.04(−0.240 to 0.320)	0.778	−1.06(−1.777 to 0.343)	0.004
Gestational weeks	0.045(0.003 to 0.086)	0.035	−0.001(−0.011 to 0.010)	0.899	0.010(−0.018 to 0.037)	0.49
Times of Pregnant						
1	ref		ref		ref	
2	0.331(−0.734 to 1.396)	0.543	−0.078(−0.353 to 0.198)	0.583	0.520(−0.194 to 1.234)	0.155
3	−0.694(−1.971 to 0.584)	0.288	−0.052(−0.384 to 0.279)	0.757	0.370(−0.487 to 1.226)	0.398
≥4	−0.447(−1.806 to 0.913)	0.520	0.010(−0.342 to 0.363)	0.953	0.734(−0.177 to 1.646)	0.116
Times of Birth						
0	ref		ref		ref	
1	−0.068(−1.007 to 0.870)	0.887	0.035(−0.208 to 0.278)	0.779	0.736(0.108 to 1.362)	0.022
≥2	−1.272(−2.768 to 0.224)	0.097	−0.039(−0.427 to 0.349)	0.843	0.717(−0.282 to 1.716)	0.161
Times of Abortion						
0	ref		ref		ref	
1	−1.30(−2.325 to −0.279)	0.013	−0.221(−0.486 to 0.045)	0.105	−0.122(−0.815 to 0.570)	0.730
≥2	−0.576(−1.752 to 0.599)	0.337	−0.115(−0.420 to 0.190)	0.461	0.049(−0.747 to 0.845)	0.904
Education Degree						
High school or blow	ref		ref		ref	
Secondary or tertiary	2.116(1.234 to 2.998)	<0.001	−0.043(−0.288 to 0.20)	0.729	0.394(−0.232 to 1.019)	0.219
Undergraduate or above	4.109(2.959 to 5.260)	<0.001	0.311(−0.008 to 0.631)	0.057	1.592(0.775 to 2.409)	<0.001
Husband’s Education Degree						
High school or blow	ref		ref		ref	
Secondary or tertiary	1.918(1.014 to 2.822)	<0.001	0.165(−0.087 to 0.417)	0.201	0.610(−0.037 to 1.257)	0.066
Undergraduate or above	4.630(3.471 to 5.789)	<0.001	0.494(0.169 to 0.817)	0.003	1.719(0.888 to 2.548)	<0.001
Monthly income of Family						
≤5000	ref		ref		ref	
5000–7000	1.729(0.512 to 2.947)	0.006	0.230(−0.107 to 0.567)	0.182	0.396(−0.465 to 1.256)	0.368
7000–10000	1.692(0.499 to 2.885)	0.006	0.290(−0.039 to 0.620)	0.086	0.992(0.148 to 1.835)	0.022
10000–20000	3.655(2.442 to 4.868)	<0.001	0.395(0.059 to 0.731)	0.022	1.606(0.748 to 2.464)	<0.001
>20000	4.628(3.108 to 6.149)	<0.001	0.454(0.033 to 0.875)	0.035	1.348(0.273 to 2.423)	0.015
Weight increment by week	−0.242(−0.742 to 0.257)	0.342	−0.087(−0.210 to 0.041)	0.185	0.087(−0.248 to 0.423)	0.61
BMI	−0.070(−0.139 to −0.001)	0.048	0.006(−0.011 to 0.023)	0.511	0.020(−0.026 to 0.066)	0.395
Career						
Teachers/Medical staff	ref		ref		ref	
Employees of public institutions	−3.725(−6.648 to −0.782)	0.014	0.030(−0.766 to 0.826)	0.941	−0.270(−2.33 to 1.792)	0.798
Company employees	−2.923(−4.544 to −1.302)	<0.001	0.050(−0.389 to 0.491)	0.821	−0.228(−1.368 to 0.911)	0.695
Worker	−5.879(−7.901 to −3.856)	<0.001	−0.258(−0.807 to 0.290)	0.357	−1.212(−2.634 to 0.210)	0.096
Farmer	−6.126(−9.213 to −3.038)	<0.001	−0.863(−1.701 to −0.024)	0.045	−1.234(−3.405 to 0.937)	0.266
Freelancer	−3.219(−4.914 to −1.523)	<0.001	−0.190(−0.650 to 0.270)	0.420	−0.444(−1.636 to 0.748)	0.466
Unemployed or others	−3.950(−5.599 to −2.300)	<0.001	−0.159(−0.607 to 0.289)	0.487	−1.166(−2.326 to −0.006)	0.050
Career Of Husband						
Teachers/Medical staff	ref		ref		ref	
Employees of public institutions	−4.000(−7.109 to −0.890)	0.012	−0.143(−0.997 to 0.711)	0.744	−1.095(−3.316 to 1.125)	0.335
Company employees	−4.509(−7.270 to −1.748)	0.002	−0.279(−1.038 to 0.479)	0.471	−1.220(−3.192 to 0.753)	0.227
Worker	−7.496(−10.344 to −4.649)	<0.001	−0.542(−1.325 to 0.240)	0.176	−2.048(−4.083 to −0.014)	0.049
Farmer	−7.679(−12.144 to −3.213)	<0.001	−0.500(−1.727 to 0.727)	0.425	−2.286(−5.465 to 0.904)	0.161
Freelancer	4.455(−7.268 to −1.641)	0.002	−0.500(−1.273 to 0.273)	0.206	−1.378(−3.388 to 0.623)	0.180
Unemployed or others	−3.714(−7.522 to 0.093)	0.057	−1.000(−2.046 to 0.046)	0.062	−0.429(−3.148 to 2.291)	0.758

**Fig. 2:**
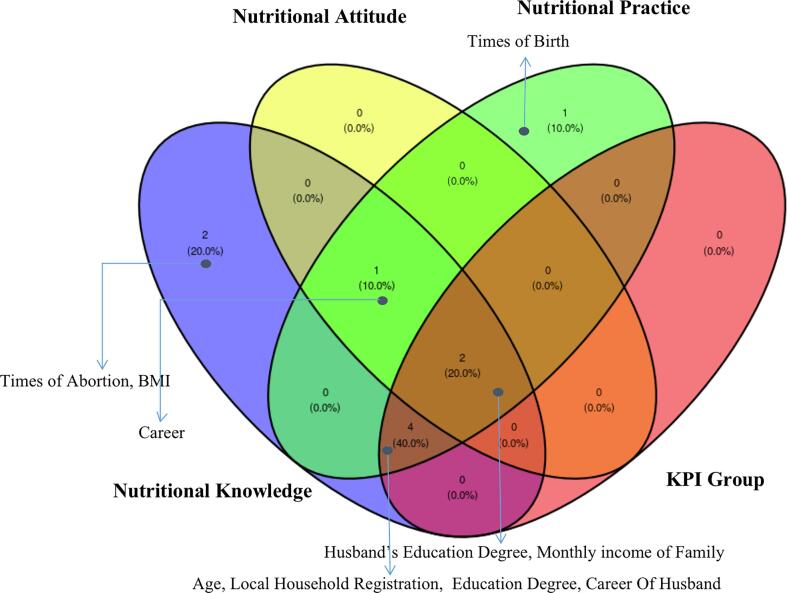
Venn plot to compare the statistically significant risk factors among Nutritional Knowledge, Attitude, Practice and KPI groups.

To explore the differences and overlap of factors across outcomes (Knowledge, Attitude, Practice and KPI), we used a Venn diagram for visualization. As shown in Fig. 2, the four circles represent 4 models. It can be seen that 2 factors were overlapping in all 4 models, namely Husband's Education Degree, Monthly income of Family. 4 factors (Age, Local Household Registration, Education Degree, Career of Husband) overlapped in 3 models (Knowledge, Practice and KPI).

### 3.5 A visualization model to locate the low-nutritional-practice pregnant

**Fig. 3:**
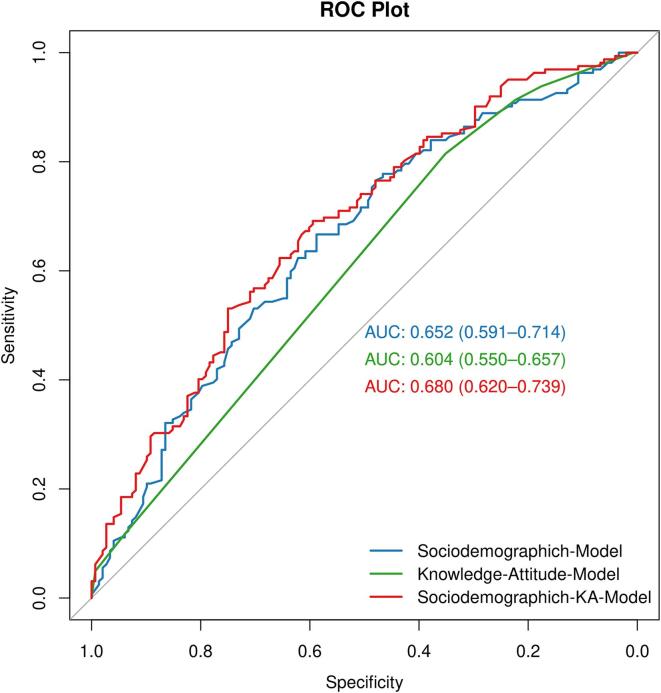
Comparison of ROC curves and AUC values when adding different batches of variables to predict the key population for intervention (KPI).

**Fig. 4:**
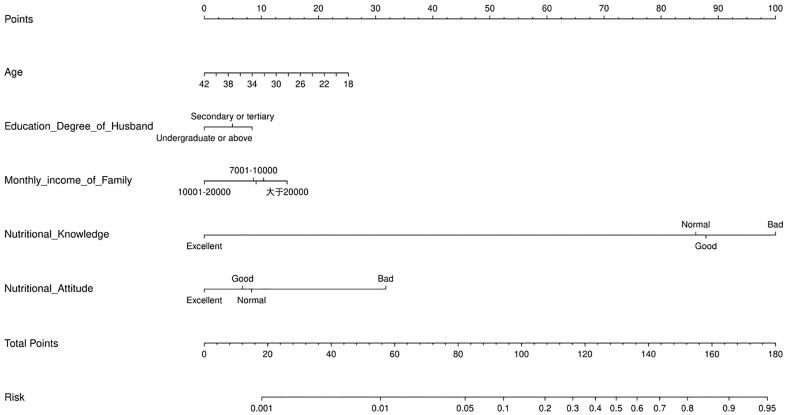
A nomogram plot to predict the key population for intervention (KPI).

It is known that sociodemographic factors can help us identify people with low nutrition practice, while knowledge and attitudes also have an impact on practice. Socio-demographic information will be easier to be obtained, knowledge and attitude will require more information entries to be filled out. Therefore, this study compares the effectiveness of only socio-demographic information or knowledge and attitude, and both combined, in identifying KPI. In the univariate analysis, we screened three sociodemographic variables, Age, Education Degree of Husband and Monthly income of Family, through a stepwise algorithm. According to Fig. 3, we drew ROC curves for each of the three scenarios, and we found that combining socio-demographics, knowledge and attitude to obtain a better AUC value (0.68). Thus, this study combines 3 socio-demographic variables as well as knowledge and attitudes to build the final model. The result of the multivariate regression analysis was shown in Table 4. The younger, the lower the husband's education, the monthly income below 5000, the poorer the knowledge and attitude, the more likely to be in need of a nutrition practice intervention. To facilitate the application of this prediction model in clinical practice, this study visualized this prediction model using the nomogram diagram. According to Fig. 4, the scores obtained for the 5 variables are summed to obtain the risk requiring intervention. It can be seen that nutrition knowledge and attitude have a greater influence on the total score.

**Table 4:** Multivariates analysis for locating the vulnerable group who needs intervention.

Variables	OR	95 %CI	*P*-value
Age	0.937	0.885–0.992	0.025
Husband’s Education Degree			
High school or blow	1		
Secondary or tertiary	0.809	0.463 ∼ 1.414	0.457
Undergraduate or above	0.596	0.267 ∼ 1.331	0.207
Monthly income of Family			
≤5000	1		
5000–7000	0.922	0.447 ∼ 1.901	0.826
7000–10000	0.896	0.438 ∼ 1.834	0.765
10000–20000	0.526	0.238 ∼ 1.166	0.114
>20000	1.291	0.460 ∼ 3.622	0.628
Nutritional knowledge			
Bad	1		
Normal	0.422	0.185 ∼ 0.964	0.041
Good	0.471	0.126 ∼ 1.756	0.262
Excellent	<0.001	<0.001∼ >100	0.781
Nutritional attitude			
Bad	1		
Normal	0.234	0.023 ∼ 2.343	0.216
Good	0.212	0.025 ∼ 1.767	0.152
Excellent	0.140	0.016 ∼ 1.263	0.080

## 4 Discussion

Many studies had shown that many sociodemographic factors influenced the practice of pregnant females, age and the level of education were the main confounding factors (Li et al., 2019). However, due to economic uncertainty, there are differences in fertility intentions and childbearing ages among females of different eras and different places (Novelli et al., 2021). For example, young females in first-tier cities tend to have a higher childbearing age than young females living in third-tier and fourth-tier cities (Riederer and Buber-Ennser, 2019). And females in the first-tier cities need to earn a living in the first-tier cities, often facing greater life pressure, which also affects their fertility intentions (Novelli et al., 2021). Most of the population in this age range (28.6 ± 4.5) was reported to face housing and employment pressures in Shenzhen and was a highly mobile population (Zhong et al., 2018). As a super-tier city with rapid GDP growth, Shenzhen's cost of living is increasing. Moreover, most are migrant workers born around 1990, who still retain the knowledge system of their parents containing unhealthy habits (Yang et al., 2015), which is quite different from that in the era of relatively abundant material resources. These groups are the builders of Shenzhen, while the most vulnerable part of Shenzhen.

As our study hypothesized, knowledge and attitude were important determinants of practice. In the correlation analysis, the practice was significantly correlated with attitude and knowledge, which is consistent with previously reported studies (Masuku and Lan, 2014). Meanwhile, results show that there was a gap between knowledge and attitude, attitude and practice, which is in line with other studies (Worsley, 2002). This phenomenon fully explained the positive attitude of respondents towards nutrition, but the reserve of nutrition knowledge restricted the transformation of nutrition attitude to nutrition practice (Worsley, 2002, Koch et al., 2021). The lack of knowledge about nutrition-related guidelines and guidance recommendations shows that while Shenzhen is developing rapidly, it is necessary to popularize basic nutritional education, especially nutrition guidelines. Fortunately, basic common knowledge such as the deficiencies in pregnancy cramps was well known. Therefore, for nutritional promotion, some specific areas should be preferentially popularized (Bonnel, 2003). In terms of nutrition attitudes, the vast majority of people are aware of the importance of nutrition, indicating that the respondents had a strong willingness to improve their nutritional status (Li et al., 2020). However, in terms of nutritional label credibility, respondents had some concerns about the credibility of nutritional labels on foods (Küster-Boluda and Vila, 2020). In nutrition practice, failure to check food nutrition labels, and poor folic acid uptake rates suggest that there is still more room for improvement in nutritional habits. In particular, most have experienced changing their food choices by checking food nutrition labels. This indicates that they trusted the information on food labels and were willing to make behavioral changes (Küster-Boluda and Vila, 2020).

Health intervention is accompanied by a cost. In comparison with interventions on the whole population, this study preferred to locate the most vulnerable group. In this study, the population with a score of less than 60% in nutrition practice was considered as the key intervention population.

Our study showed several sociodemographic factors are significantly related to whether they are the key population. A study in Swaziland (Masuku and Lan, 2014) also reported that education level, monthly income, and employment status were associated with the practice. Therefore, the focus of intervention can be located on these sociodemographic factors, especially on groups with lower education and lower monthly income. It is worth noting that, unlike studies in other areas, part of pregnant females in Shenzhen are often floating population, so their residence location is also an important factor, and the intervention group should be more likely to be those whose household registries are not in Shenzhen (Campos et al., 2016, Mu and De Brauw, 2015). Knowledge, attitude, and practice have a significant correlation, but the influencing factors of these three outcomes were also slightly different. Age, household registry and education level were significant in nutrition knowledge and nutrition practice, but not in nutrition attitudes, indicating that respondents of different ages, different household registries and different education levels were relatively consistent in nutrition attitudes, but younger and non-local household registry, less educated respondents influenced nutrition practice due to limitations in nutrition knowledge (Bundala et al., 2020). The transformation of nutrition practices due to knowledge limitations in this group confirms the benefits for nutrition knowledge intervention. The educational level of the respondents' husbands was significantly associated with nutrition knowledge, attitude, and practice, indicating that mutual education between husbands and wives also affected knowledge, attitude, and practice (Bundala et al., 2020), which indicated the effectiveness of peer education, which was consistent with previous reports (Duncanson et al., 2014, Story et al., 2002). The household's monthly income was also an important factor influencing the nutrition knowledge, attitude and practice of the respondents (Behrman and Deolalikar, 1987). From the results of the Venn diagram, it can be seen that four factors (Age, Local Household Registration, Education Degree, and Career of Husband) are related to the nutrition knowledge and practice of the respondents, but not to the nutrition attitude. The population related to these four factors may be affected by the lack of knowledge, so health education intervention should be carried out to make up for the lack of knowledge and achieve the purpose of practice transformation.

In this study, age, husband's education, monthly family income, nutrition knowledge, and nutrition attitude were selected as the main factors for screening the KPI. In the multivariate regression model, the husband's education level and monthly household income are not statistically significant, but they were still selected into the regression model because the addition of these factors makes the model more robust and the regression coefficient more accurate. According to the nomogram plot, the younger, the lower the education level of the husband, and the lower the monthly household income (<20,000 RMB), the lower the nutrition knowledge and attitude of the population are more likely to be the population who would benefit from intervention. For the evaluation of the model, the AUC value increased from 0.652 to 0.68 after adding nutrition knowledge and attitude variables, indicating that nutrition knowledge and nutrition attitude variables had some effect on the key interventions.

It is important to note that the results of this study are mainly from a cross-sectional study and there is no evidence of the causal relationship between knowledge, attitude and practices. This study can only show that there is some correlation between each other. But the bidirectionality between them is an interesting topic and further studies may consider using longitudinal data to examine the bidirectionality between KAP. Furthermore, external validity is a limitation of this study because the causal relationship between the variables could not be determined and thus the results of this study could not be extrapolated to other populations. It is expected that subsequent studies will explore this issue in more depth and contribute more to the solution of nutritional health problems of pregnant females.

## 5 Conclusion

There was a gap between nutritional knowledge and attitude, attitude and practice. Age, household registry, education level (including pregnant females and their husbands), monthly income, and nutrition knowledge were important predictors of nutrition practices among pregnant females in Shenzhen. This study presents a predictive model to predict key populations who would benefit most from intervention, but its causal relationship needs further studies.

## 6 Limitations

The results of this study may vary across regions, but provide evidence for other similar studies. Face-to-face interviews may lead to reactions expected by society.

## CRediT authorship contribution statement

ZD and JYF - Conceptualizaiton, Funding Acquisition, Investigation, Methodology. WCW, SMZ and JYF - Data curation, Formal Analysis. WCW and JYF- Writing. ZD and JYF - Validation.

## Declaration of Competing Interest

The authors declare that they have no known competing financial interests or personal relationships that could have appeared to influence the work reported in this paper.

## Data Availability

Data will be made available on request.
