# Supplementary material for: Nutritional knowledge, attitude and practices among pregnant females in 2020 Shenzhen China: A cross-sectional study
Source: Prev Med Rep. 2023 Feb 18;32:102155. doi: 10.1016/j.pmedr.2023.102155 (PMC9975685; doi:10.1016/j.pmedr.2023.102155)
Supplement: Supplementary data 1 [file mmc1.docx]

Supplementary material

Appendix 1 Distribution of nutritional knowledge-related response of pregnant (N=310)

| **Variable** | **True** | | **Partially True** | | **False** | |
| --- | --- | --- | --- | --- | --- | --- |
|  | **N** | **%** | **N** | **%** | **N** | **%** |
| Heard about Nutritional Dietary Guidelines, | 136 | 43.9 | - | - | 174 | 56.1 |
| Which food provide energy | 156 | 50.3 | - | - | 154 | 49.7 |
| Which food provide high-quality protein | 157 | 50.6 | 76 | 24.5 | 77 | 24.8 |
| Carbohydrates and sugars are one thing | 129 | 41.6 | - | - | 181 | 58.4 |
| Whether milk is iron-rich | 163 | 52.6 | - | - | 147 | 47.4 |
| What nutrients are lacking causing cramps during pregnancy | 282 | 91.0 | - | - | 28 | 9.0% |
| Which nutrients is associate with high blood pressure | 75 | 24.2 | - | - | 235 | 75.8 |
| Whether to know the food packaging is subject to mandatory labeling of food nutrition labels | 112 | 36.1 | - | - | 198 | 63.9 |
| Contents of food nutrition labels | 2 | 0.6 | 136 | 43.9 | 172 | 55.5 |
| Know the NRV | 22 | 7.1 | - | - | 288 | 92.9 |
| Understand the food nutrition labels | 39 | 12.6 | 180 | 58.1 | 91 | 29.3 |

Appendix 2 Distribution of nutritional attitude-related response of pregnant (N=310)

| **Variable** | **Strongly Yes** | | **Yes** | | **Partially Yes** | | **No** | |
| --- | --- | --- | --- | --- | --- | --- | --- | --- |
|  | **N** | **%** | **N** | **%** | **N** | **%** | **N** | **%** |
| Nutritional Knowledge important to you | 240 | 77.4 | - | - | 69 | 22.3 | 1 | 0.3 |
| Willing to change the eating habits | - | - | 307 | 99 | - | - | 3 | 1.0 |
| Is the information on nutritional food label credible | 50 | 16.1 | 187 | 60.3 | 65 | 21.0 | 8 | 2.6 |
| Necessary to show the food label | - | - | 304 | 98.1 | 4 | 1.3 | 2 | 0.7 |

Appendix 3 Distribution of nutritional practice-related response of pregnant (N=310)

| **Variable** | **N (Percentage)** |
| --- | --- |
| Take folic acid-rich foods or folic acid supplements daily |  |
| Yes | 200(64.5%) |
| No | 110(35.5%) |
| Drink milk or calcium tablets daily |  |
| Yes | 226(72.9%) |
| No | 84(27.1%) |
| Do not eat fried smoked or carbonated food |  |
| Yes | 83(26.8%) |
| No | 227(73.2%) |
| There have been experiences of changing food choices because of food labels |  |
| Yes | 178(57.4%) |
| No | 132(42.6%) |
| Whether to look at the food nutrition label when selecting food |  |
| Always | 48(15.5%) |
| Often | 93(30.0%) |
| Sometimes | 128(41.3%) |
| Rarely | 41(13.1%) |

Appendix 4 Results of Pearson’s correlation between nutritional KAP and sociodemographic variables

| **Variable1** | **Variable 2** | **Correlation Coefficient (95%CI)** | ***P* value** |
| --- | --- | --- | --- |
| Nutritional knowledge | Nutritional attitude | 0.194(0.085~0.303) | <0.001 |
| Nutritional knowledge | Nutritional practice | 0.316(0.217~0.415) | <0.001 |
| Nutritional knowledge | Age | 0.178(0.068-0.289) | 0.002 |
| Nutritional knowledge | Gestational weeks | 0.120(0.004-0.236) | 0.035 |
| Nutritional knowledge | Weight increment by week | -0.054(-0.176-0.068) | 0.342 |
| Nutritional knowledge | BMI | -0.112(-0.229-0.004) | 0.048 |
| Nutritional attitude | Nutritional practice | 0.330(0.232-0.428) | <0.001 |
| Nutritional attitude | Age | 0.055(-0.066-0.177) | 0.334 |
| Nutritional attitude | Gestational weeks | -0.007(-0.134 - 0.119) | 0.899 |
| Nutritional attitude | Weight increment by week | -0.075(-0.195-0.044) | 0.185 |
| Nutritional attitude | BMI | 0.037(-0.086-0.161) | 0.511 |
| Nutritional practice | Age | 0.214(0.107-0.321) | <0.001 |
| Nutritional practice | Gestational weeks | 0.039(-0.084-0.163) | 0.490 |
| Nutritional practice | Weight increment by week | 0.029(-0.095-0.153) | 0.610 |
| Nutritional practice | BMI | 0.048(-0.074-0.171) | 0.395 |
| Age | Gestational weeks | 0.017(-0.107-0.143) | 0.753 |
| Age | Weight increment by week | 0.040(-0.083-0.163) | 0.481 |
| Age | BMI | 0.009(-0.117-0.136) | 0.871 |
| Gestational weeks | Weight increment by week | -0.179(-0.289 - -0.069) | 0.002 |
| Gestational weeks | BMI | -0.039(-0.163 - 0.084) | 0.490 |
| Weight increment by week | BMI | -0.014(-0.141 - 0.111) | 0.793 |
